# Erie County Medical Society

**Published:** 1867-06

**Authors:** T. M. Johnson

**Affiliations:** Secretary


### Erie County Medical Society.

The semi-annual meeting of the Erie County Medical Society, was held at the rooms of the Buffalo Medical Association on the 11th inst. The meeting was well attended, and the usual routine of business of such meetings transacted. The following gentlemen were elected to membership upon compliance with the By-Laws: Drs. Conrad Diehl, Byron H. Daggart, Charles F. A. Nichell, Gustavus E. Mackay, Milton G. Potter, of Buffalo; and John C. Potter, of Lancaster.

T. M. Johnson, M. D., Secretary.

